# One size fits all?: A simulation framework for face-mask fit on population-based faces

**DOI:** 10.1371/journal.pone.0252143

**Published:** 2021-06-16

**Authors:** Tomas Solano, Rajat Mittal, Kourosh Shoele

**Affiliations:** 1 Department of Mechanical Engineering, Joint College of Engineering Florida State University-Florida A&M University, Tallahassee, Florida, United States of America; 2 Department of Mechanical Engineering, Johns Hopkins University, Baltimore, MD, United States of America; 3 School of Medicine, Johns Hopkins University, Baltimore, MD, United States of America; Istituto Italiano di Tecnologia, ITALY

## Abstract

The use of face masks by the general population during viral outbreaks such as the COVID-19 pandemic, although at times controversial, has been effective in slowing down the spread of the virus. The extent to which face masks mitigate the transmission is highly dependent on how well the mask fits each individual. The fit of simple cloth masks on the face, as well as the resulting perimeter leakage and face mask efficacy, are expected to be highly dependent on the type of mask and facial topology. However, this effect has, to date, not been adequately examined and quantified. Here, we propose a framework to study the efficacy of different mask designs based on a quasi-static mechanical model of the deployment of face masks onto a wide range of faces. To illustrate the capabilities of the proposed framework, we explore a simple rectangular cloth mask on a large virtual population of subjects generated from a 3D morphable face model. The effect of weight, age, gender, and height on the mask fit is studied. The Centers for Disease Control and Prevention (CDC) recommended homemade cloth mask design was used as a basis for comparison and was found *not* to be the most effective design for *all* subjects. We highlight the importance of designing masks accounting for the widely varying population of faces. Metrics based on aerodynamic principles were used to determine that thin, feminine, and young faces were shown to benefit from mask sizes smaller than that recommended by the CDC. Besides mask size, side-edge tuck-in, or pleating, of the masks as a design parameter was also studied and found to have the potential to cause a larger localized gap opening.

## 1 Introduction

During the COVID-19 pandemic, wearing face masks is the new status quo, and it has become apparent that the fit of the mask is important. In the early stages of the pandemic, face masks were primarily used as a barrier to small droplets that could carry the virus. Recently, however, scientists have urged public-health authorities to acknowledge the potential for airborne transmission of the novel SARS-CoV-2 coronavirus [1]. While there is still a lot that is unknown about the transmission of the SARS-CoV-2 virus, it is evident now that like its predecessor, SARS-CoV-1, airborne transmission is a significant mode of transmission [2–4]. Airborne transmission happens when a susceptible person inhales microscopic bio-aerosols in the air which are generated from a respiratory event such as a cough, sneeze, or even just breathing and talking [2, 5]. While larger droplets (≥100*μm*) reach the ground within a second, aerosols can linger in the air for hours, increasing the probability of a susceptible person coming in contact with the virus [6, 7]. For this reason, mask fit is important.

Experimental studies with human subjects and manikins show that mask usage can limit the droplet and airborne transmission of various infections to and from the wearer [8–16]. Air leakage has been observed around the perimeter of the mask where it does not make a seal with the face, reducing the effectiveness of the mask [6, 17]. Perimeter leakage is caused by loose or improper fitting face masks and can be significantly impacted by facial features [18–21]. A recent study found that seemingly insignificant facial features have an impact on the fitting of the mask on the face and concluded that 3D models could be used to assess mask fit in relation to the subtle changes in facial topology [17]. While proper-fitting of respirators on a face has always been stressed for effective filtering of all contaminants, there is a lack of knowledge on how important the fit of homemade cloth and simple surgical masks is. Surgical masks are primarily designed for outward protection from droplets, not aerosols, and therefore, the fit is much looser. Homemade masks made from cotton or similar fabrics are likely to be even looser, allowing for more leakage. These looser-fitting masks are more susceptible to perimeter leakage and, therefore, not as effective against aerosols.

In a study comparing the effectiveness of different face masks, homemade masks were shown to be half as effective as surgical masks and 50 times less effective than an FFP2 mask (similar to an N95 respirator; filters 94% of particles larger than 0.3 microns). These effects were even more pronounced amongst the children subjects, likely due to an inferior fit on their smaller faces [15]. More recently, Verma et al. experimentally explored the effect of different mask types by visualizing the respiratory jets and observed leakage through the perimeter of the mask [22]. The study reported that both the mask material and fit have an important impact on the mask’s effectiveness, with all masks tested showing leakage from the top of the mask due to poor fitting. The studies by Oestenstad et al [19] and Oestenstad and Bartolucci [20] used a fluorescent tracer to identify leak location and shape on subjects wearing half-mask respirators. They tested the effect of gender, race, respirator brand, and facial dimensions and found that facial dimensions were significantly correlated to the leakage location. Tang et al. studied the jet generated from coughing and the effect of wearing surgical masks or N95 respirators [23]. They found that a surgical mask effectively blocks the forward momentum of the cough jet, but the loose fit of the mask allows air leakage around the perimeter of the mask primarily through the top and sides. Lei et al. used a headform finite element model to study the leakage locations of an N95 respirator and show that the most leakage occurs along the top perimeter of the mask near the nose [24]. From previous studies, we can conclude that although it is understood that mask fit is important and affected by facial features, it is not clear yet how and which features impact the fit. The simulations of face-masks and population-based headform models have the potential to accurately estimate the location and amount of leakage for different facial structures. These 3D models can also be leveraged to systematically explore the effect of different facial features in order to design better masks.

A strong argument can be made for the importance of accurate mask-fit models for the prediction of virus transmission. Mathematical frameworks that model the spread of a virus in public spaces, cities and entire countries must take into account the rate of transmission to and from each member in the population. The rate of transmission of a virus is dependent on the severity of the virus itself, population density, and mask effectiveness [4, 9, 25, 26]. The parameters relating to the effectiveness of face-masks in these transmission models vary significantly, drastically affecting the results. Eikenberry et al. included the effect of mask usage in their model based on the inward and outward efficiencies of face-masks. They cite a wide range of mask efficiencies, ranging from 20 to 80%, derived from experimental studies [14–16]. The experimental studies of course are limited in the number of subjects tested, with all experimental studies mentioned previously having less than 25 subjects and in some cases as few as 7 subjects tested, from which mask efficiencies were calculated. The limitation of the number of subjects and range of facial features in experimental studies means that statistically significant results from which we can derive correlations of mask fit and face types or topology are hard to come by, if not nonexistent. Eikenberry et al. illustrate the significance of proper estimation of mask effectiveness by showing that the effective transmission rate decreases linearly proportional to the mask efficiency such that masks with 20% and 80% efficiency decrease effective transmission rate by approximately 20% and 80% respectively [9]. Such disparities in mask efficiency can lead to less than reliable models of the spread of the virus. Mittal et al. recently proposed a new transmission model, the COVID-19 airborne transmission (CAT) inequality. The model was designed for simplicity so that it can serve as a common scientific basis and also be understood by a more general audience [4]. Like Eikenberry’s and Briennen’s models, the CAT inequality accounts for the protection afforded by face coverings. The effect of face-masks in the CAT inequality is primarily based on the filtration properties of the material. All of these transmission models attempt to predict the spread of viruses, which requires a proper statistical model to account for the effectiveness of face-masks. Understanding and developing reliable models for the effectiveness of face coverings based on not only the fabric material but also the fit can lead to better transmission models.

Accurate characterization of mask effectiveness due to individual fit goes beyond reliable transmission models and more directly affects the general public. The CDC has provided design guidelines for home-made masks, but as we will show in this study the recommended mask design, or any single mask design, may not be optimally effective for the many different facial structures inside a population. That is to say, one size *does not* fit all. Instead, to ensure the effectiveness of the mask, particular sizes and designs should be recommended for several distinct population categories. Here, we develop a framework to study the effect of the varying facial features in a large population on the mask fit and efficacy. The framework provides a systematic platform on which many different mask designs can be quickly tested on a larger population of faces than could otherwise be achieved under very extensive and costly traditional experimental tests. This study aims to provide a framework to develop better mask designs and provide motivation as to why mask fit and mask design should be studied at an individual level. Specifically, we look at the leakage from a simple home-made mask design as recommended on the CDC website [27], and show how the size and simple adjustment of face-masks can affect the mask leakage based on the facial features. The goal is to illustrate the practical application of such a framework and identify potential discriminative metrics for future studies. Here, different faces are categorized based on the subject’s weight, age, gender, and height. The proposed framework can further be extended to different facial features and more complex mask designs. However, here, we introduce the methodology and its application for studying the efficacy of rectangular homemade cloth masks.

## 2 Methodology

The performance of the face-masks is highly dependent on the properties of the mask as well as the fit of the mask on a given face. Here, we employ three-dimensional morphable models of the human face to account for gender, age, and other body-habitus-associated variabilities in face morphologies and conduct mask-deployment simulations for a large “virtual cohort” of individuals. The goal is to quantify the mode of perimeter opening and study how the mask leakage is changing with a population’s facial features. The components of the computational model are represented below.

### 2.1 Virtual cohort of faces

**Fig 1:**
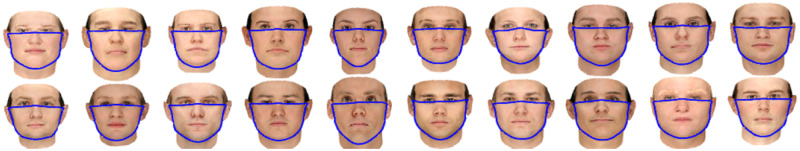
Twenty sample random realizations of the base face category from the virtual cohort.

The morphable model is based on the Basel Face Model (BFM) [28], a publicly available database that includes face scans of more than 100 males and 100 females ranging from 8 to 62 years old with weight ranging from 40 to 123 kg. Since the BFM database is pre-processed with principal component analysis (PCA), we will use the low dimensional PCA subspace to create realistic in-silico face realizations [29]. Fig 1 shows sample realizations of a face based on subspace synthesis. In addition, using identified feature vectors associated with weight, gender, age, and height, each realization can further be modified toward a particular shape. A similar morphing mesh is used for different faces, and separate regions and landmarks of the lips, ears, nose, and eyes are identified on the model. These landmarks are utilized to establish the mask position for a given face. Due to the PCA subspace, feature vectors associated with any number of relevant face characteristics beyond the ones studied here can be identified and systematically correlated to the mask fit. In addition, different facial expressions can also be modeled similarly.

### 2.2 Deployment modeling

**Fig 2:**
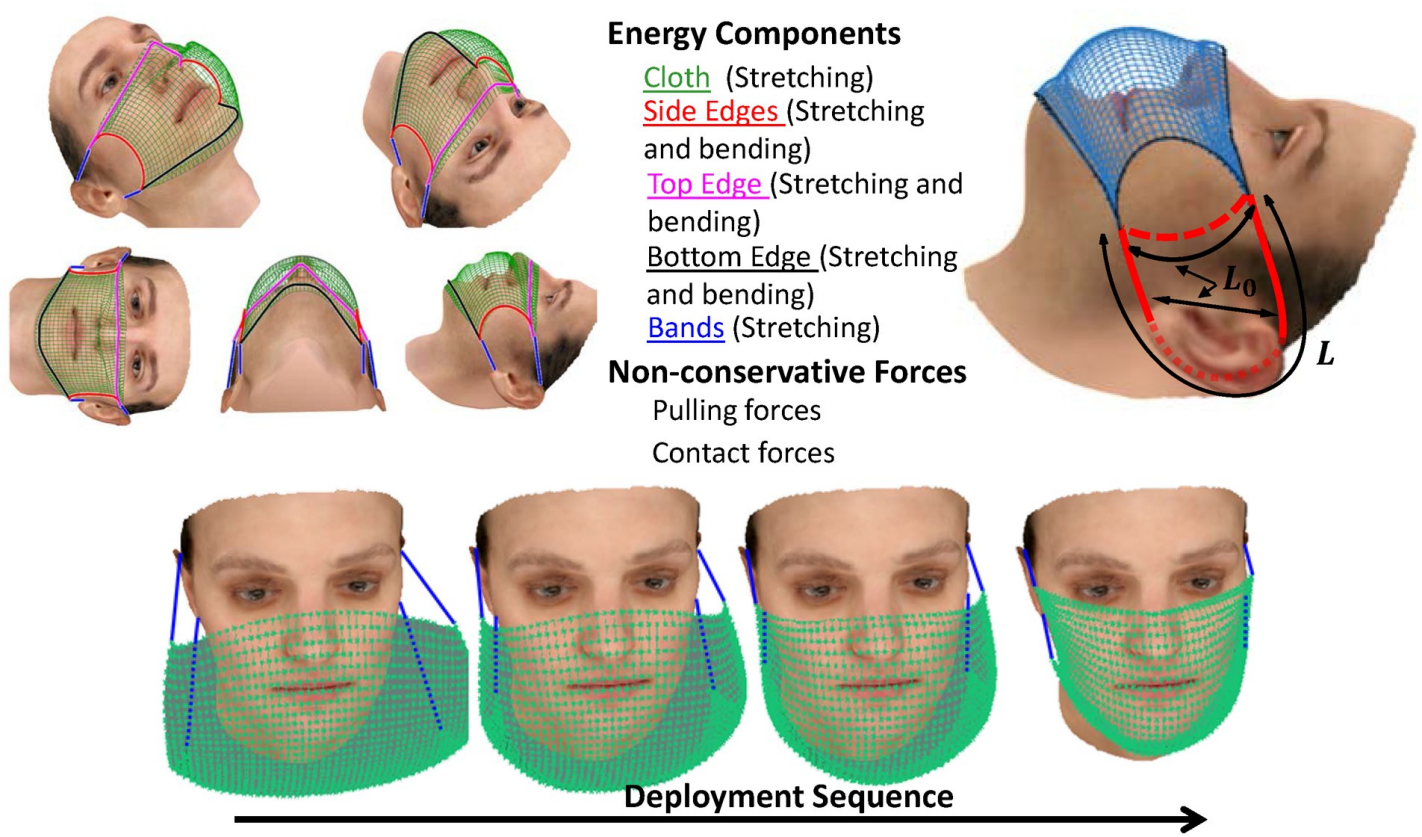
(a) Major structural components and interaction forces of a cloth-type face-mask and their placement of the model, (b) the initial and deformed lengths of the boarder, (c) the deployment sequence.

A quasi-static model is employed for the deployment of a mask on a given virtual face. In the simulation, the mask is initially placed in front of the face with elastic bands wrapped around the ears but with zero tension. The resting length of the band gradually decreases from the initial length to its final value during the initial transient phases and for each stage, the intermediate quasi-static equilibrium position of the mask is calculated from the model in section 2.3. The procedure is continued until the mask rests in the final configuration on the face (Fig 2c). The procedure is repeated for different face realizations, and for each realization, the face is systematically modified in 8 directions, namely, thinner or heavier, younger or older, more feminine or more masculine, and shorter or taller features. For each case, the ensemble statistics of a particular group are calculated and cross-compared.

### 2.3 Fabric mask model using minimum energy concept

Because of the mask’s small flexural stiffness, the band’s geometrical constraints, and the contact between the mask and the face, the mask could have local buckling as wrinkles and slacks on its surface [30]. To account for all of these effects, a detailed multi-scale approach is required to represent diversely scaled elements from the dominant fibers in the mask to the interaction between human facial tissue and the mask surface. Here we use the minimum energy concept as a unified principle of mechanics that works across all scales and governs the position of the mask on the face. In this method, the total elastic energy of the system is expressed as,

$$\begin{array}{c} E_{t}\left(X\right)=E_{cloth}^{s}+E_{border}^{s}+E_{border}^{b}+E_{band}^{s}, \end{array}$$ (1)

where $E_{cloth}^{s}$ is the extensional elastic energy stored in the cloth, $E_{border}^{s}$ is the tension and compression energy in the border strip around the cloth mask, $E_{border}^{b}$ is the stored bending energy in the border strip around the cloth, and $E_{band}^{s}$ is tension energy stored in the connecting bands (refer to Fig 2a for a depiction of these effects).

The cloth is assumed to be made up of two orthonormal fiber bundles where their extensional elastic energy is an order of magnitude larger than its in-plane shear and bending energies. This assumption is justified as the regular cloth masks can be modeled as thin membranes that could easily undergo localized buckling and show negligible bending stiffness. Moreover, to account for the wrinkling effect, the energy associated with the area change of the mask is not considered. Instead, the extensional elastic energy stored in the cloth is made up of the stored energy of a group of initially orthonormal fibers according to,

$$\begin{array}{c} E_{cloth}^{s}\;=\;\int_{0}^{L_{1}}\int_{0}^{L_{2}}W_{cloth}^{s}ds_{1}ds_{2}, \end{array}$$ (2)

where *L*_1_ and *L*_2_ are initial unloaded edge lengths of the cloth mask. Here, $W_{cloth}^{s}$ is the strain energy density defined as,

$$\begin{array}{c} W_{cloth}^{s}\;=\;\frac{E_{clo}}{4(1+\nu_{clo})}\;(I_{1}-1), \end{array}$$ (3)

where *E*_*clo*_ is the elastic modulus and *ν*_*clo*_ is the Poisson ratio of the cloth respectively, and *I*_1_ = 2(*D*_11_+*D*_22_) is the first invariant of Green strain tensor defined as,

$$\begin{array}{c} D_{ij}=\frac{1}{2}(\frac{\partial X}{\partial s_{i}}\cdot \frac{\partial X}{\partial s_{j}}-\frac{\partial X^{0}}{\partial s_{i}}\cdot \frac{\partial X^{0}}{\partial s_{j}}), \end{array}$$ (4)

with **X** and **X**^0^ are the current and initial position of the cloth, respectively.

Similarly, $E_{border}^{s,b}$ is defined as

$$\begin{array}{c} E_{border}^{s,b}\;=\;\int_{0}^{L_{b}}W_{border}^{s,b}ds_{b}, \end{array}$$ (5)

where $L_{b}=\sum_{k=1}^{4}L_{k},$ is the summation of the length of all four edges of the cloth mask. Here, if **X**(*s*) and **X**^0^(*s*) are the coordinates of the border in the reference and deformed configurations respectively, we can define the attached coordinate system to the border in its deformed configuration using its tangent vector $\tau =\frac{X_{,s}}{∥X_{,s}∥}$, binormal vector $b=\frac{\tau \times X_{,ss}}{∥\tau \times X_{,ss}∥}$ and normal vector $n=\frac{b\times X_{,s}}{∥b\times X_{,s}∥}$. A similar definition is also used for the reference position of the border. The extensional strain energy density functions can then be expressed as

$$\begin{array}{c} W_{border}^{s}=\frac{A_{bor}E_{bor}}{2(1+\nu_{bor})}(\frac{\partial X}{\partial s}\cdot \frac{\partial X}{\partial s}-\frac{\partial X^{0}}{\partial s}\cdot \frac{\partial X^{0}}{\partial s}). \end{array}$$ (6)

The bending strain energy density function is approximated as

$$\begin{array}{c} W_{border}^{b}=\frac{1}{2}E_{bor}I_{bor}{\left(\kappa -\kappa^{0}\right)}^{2}, \end{array}$$ (7)

where *κ* and *κ*^0^ are the curvature in the direction **n** and **n**^0^, respectively. Here, the initial curvature of the border is chosen to be zero. The curvature can be related between local sets of three consecutive discrete nodes **X**_*i*−1_, **X**_*i*_, **X**_*i*+1_ along the rod according to [31],

$$\begin{array}{c} \kappa =\frac{2\sin\theta }{X_{i+1}-X_{i-1}}, \end{array}$$ (8)

where *θ* is the angle between two consecutive segments of the line and is defined as,

$$\begin{array}{c} \theta \;=\;\arccos(\frac{∥X_{i}-X_{i-1}{∥}^{2}+∥X_{i+1}-X_{i}{∥}^{2}-{∥X_{i+1}-X_{i-1}∥}^{2}}{2∥X_{i}-X_{i-1}∥∥X_{i}-X_{i-1}∥}). \end{array}$$ (9)

The energy contribution from the stretching band is also defined similarly to $E_{border}^{s}$, with the extensional stiffness of $\frac{A_{ban}E_{ban}}{1+\nu }$ and the unstretched length of *L*_0_. (Fig 2b).

In addition to internal energy action, the non-penetration contact force between the mask and the facial tissue is represented with non-conservative forces, **f**_*t*_ = **f**_contact_, acting on the mask surface. Here, we assume soft contact between the face and the mask, in which *f*_contact_ is defined as,

$$\begin{array}{c} f_{\text{contact}}\;=\;\{\begin{array}{cc} k_{con}\;∥X-X_{con}∥{\;n}_{con} & (X-X_{con})\cdot n_{con}<0\\ 0 & \text{Otherwise} \end{array} \end{array}$$ (10)

where **X**_*con*_ is the closest point on the face to point **X** on the mask, and **n**_*con*_ is the outward normal to this point. The contact stiffness between the skin and the mask is represented by *k*_*con*_.

By relating the internal forces of the mask to the derivatives of the energy density function, nonlinear sets of equations are obtained for the node placements *X*_*k*_ = (*x*, *y*, *z*)_*k*_ in the discrete model of the mask. The resulting equations are solved to find the equilibrium position at a given deployment stage. Moreover, since the equilibrium shape is slowly modified from the previous deployment stage,Â **X**^*n*−1^, a linearized equilibrium equation is derived for *δ* **X** = **X**^*n*^ − **X**^*n*−1^, and is employed as a preconditioner to accelerate the convergence of the solution. This is done by defining spatial virtual work in terms of virtual velocity, *δ* **v**(**X**), and a discretized solution of **X**, *ϕ*(**X**), obtained from the discrete models of the mask, border and band. The equilibrium equation is solved iteratively to find the new position **X**^*n*^ from **X**^*n*−1^ using the projective dynamics methodology [32].

**Fig 3:**
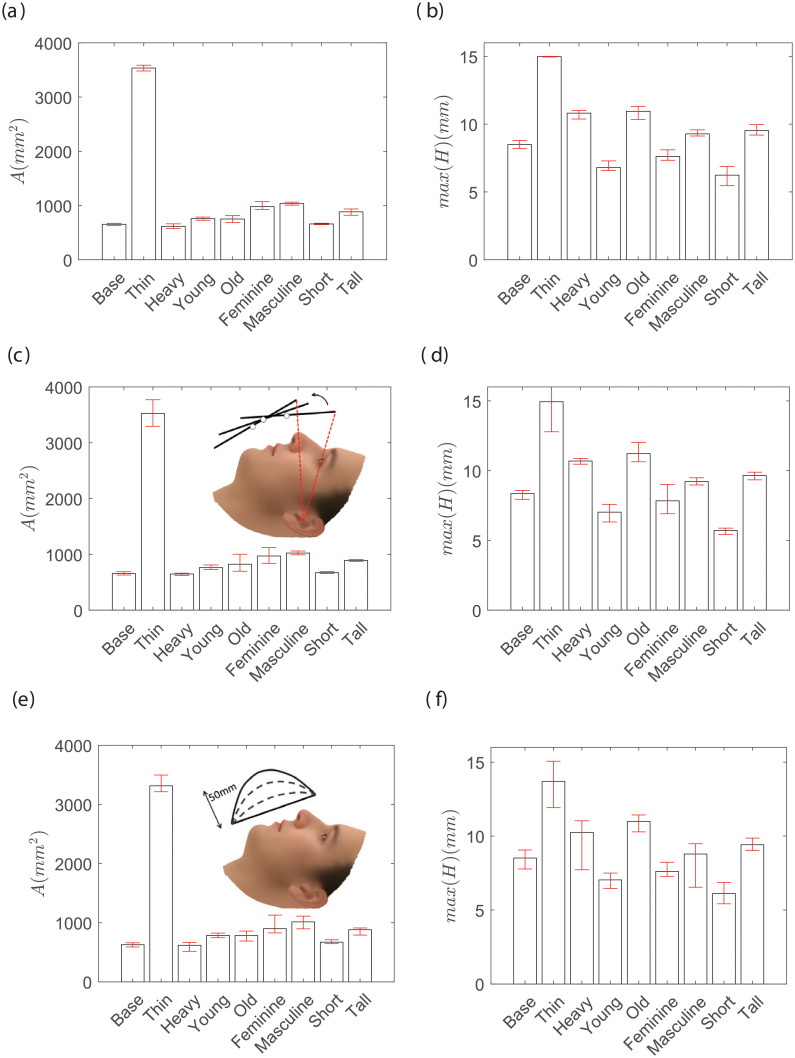
The sensitivity of total leakage area (a, c, e) and maximum gap distance around the perimeter of the mask (b, d, f) for varying range of Young Modulus, *E*_*clo*_ = *E*_*bor*_ = [7–1.3] MPa and $E_{clo}^{Base}[30-50]\text{MPa}$ (a, b), position of the mask during deployment from the top edge just covers the nose and fully blocks the vision (c, d), and the initial bulge in the middle of the mask from 0–50 mm (e, f). All the cases are done for the medium mask size with *L*_0_ = 5.5 in, *W* = 9 in and the tuck-in ratio = 0.5.

A sensitivity study was carried out to determine how the initial placement of the mask and the elastic modulus of the material affect the final fit. Fig 3a and 3b show the sensitivity for elastic modulus for all the face categories included in this study. The elastic modulus of cloth and band were varied between 7–13 MPa and *E*_*ban*_ is varied between 30 and 50 MPa, in the range of typical cloth material properties. The total leakage area (*A*) and maximum gap distance (*max*(*H*)) show no significant effects. The initial position of the mask is varied such that the top edge of the mask positions between its extreme conditions. The lowest acceptable position is the top edge is placed on the nose tip and the highest placement is when the mask covers the vision of a subject. Varying the initial position of the mask also does not result in a significant change of *A* or *max*(*H*). Finally, we tested if the initial bulge in the mask affects the leakage from the sides and found no significant effect from such effect too. Therefore, all subsequent simulations are initialized with the mid nominal parameters. The mask center is assumed to be normally approaching the mid point between the mouth and nose. We choose *E*_*clo*_ = *E*_*bor*_ = 10 MPa and Young’s modulus of the band is chosen to be 4 times stronger with *E*_*ban*_ = 40 MPa. All the Poisson ratios are fixed at 0.3 [33], which is tested (not shown here) and found not to change the observed behavior. The thickness of the cloth mask is chosen to be 0.5mm, and the band is made up of 0.5in folded cloth fabric following the recommendation of the CDC for the short edges [27]. The stretching band is assumed to be circular with a diameter of 1mm and its initial length is equal to the length of the ear. These parameters are chosen to be close to typical cotton fabric and elastic band. In addition, it is assumed that the contact stiffness between the mask and different part of the face is similar at every location on the face, with the contact stiffness *k*_*con*_ = 1Mpa, in the range of skin (0.6 MPa) and thick muscles (∼0.8 MPa) [34, 35]. These parameters have been checked to be sure that their value does not have a significant effect on the results. Also, the cloth mask is discretized with Δ*s* = 2*mm*, and grid refinement studies have been performed to ensure that the simulation results are independent of the grid sizes.

## 3 Results

### 3.1 Effect of mask size and side tuck-in ratio

**Fig 4:**
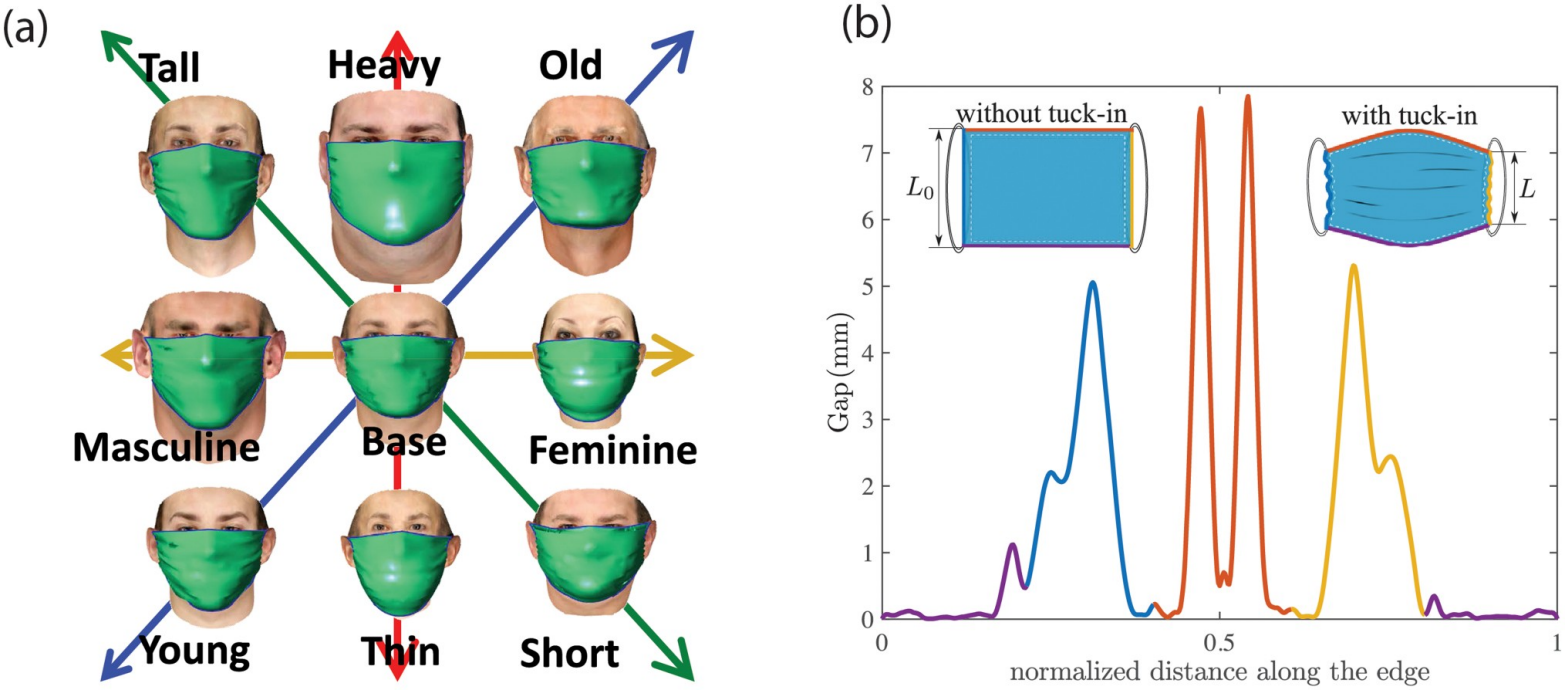
a) Sample simulation results of deploying a face-mask on a base realization face and its modification along each feature axes. b) A sample profile of leakage opening around the perimeter of the mask in the clockwise direction starting from the middle of chin side. The mask edges are named as the nose side (Area 1), cheek side (Area 2) and chin side (Area 3) and are marked with their corresponding colors in the inset figures. The inset figures also show the definition of tuck-in ratio *α*_*T*_ = *L*/*L*_0_ in the current study.

Here, the leakage area for a rectangular cloth face-mask for three different sizes and tuck-in conditions of the side edges is explored. The tuck-in of the mask is mathematically modeled as the gradual reduction of the unstretched length of side edges from the initial length (*L*_0_) to *L* = *α*_*T*_ *L*_0_, where *α*_*T*_ is named as the tuck-in ratio (Fig 4). The tuck-in can also be thought of as pleating the sides of the mask such that the side edges become shorter than the original length as shown in the inset of Fig 4b. The mask shape and size are chosen based on the guideline by CDC on how to sew cloth face coverings [27]. From the guideline, the baseline case is selected to be a mask with *L*_0_ = 5.5*in* and *W* = 9*in* (will be referred to as medium mask), with *α*_*T*_ = 0.5. Two other sizes of *W* = 8*in* (hereafter will be referred to as small mask) and *W* = 10*in*. (will be referred to as large mask) with the same aspect ratio are also tested to account for the variation in the mask size.

To ensure the ensemble statistics are sufficient to reach a confident inference from the simulations, 150 random subjects are selected from the virtual cohort of faces and for each subject, 8 modified configurations are generated to systematically explore how the facial features affect the leakage area around the perimeter of a mask. The modification to each random face is done along one feature direction at a time. The feature vectors in this study are restricted to weight (thin to heavy), age (young to old), gender (feminine to masculine), and height (short to tall) as shown in Fig 4a. The selection is based on the availability of the prior database. Other important features of faces such as race are not considered and will be explored in future work, as explained in the conclusion section.

**Fig 5:**
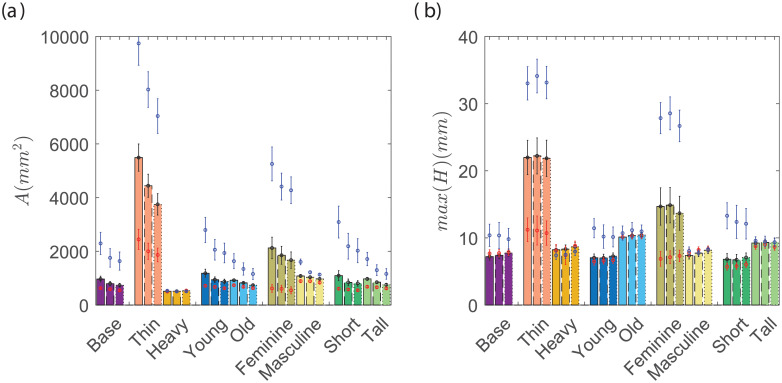
a) Average perimeter leakage opening area, b) the maximum gap distance. Bars are for medium (CDC recommended) mask. Red and blue dots represent the small and large masks, respectively. The tuck-in ratio of 0.7 is shown with solid bars, 0.5 with dashed bars, and 0.3 with dotted bars. The standard deviation of each data point is displayed with a line.

Fig 5a shows the statistical mean value of the cumulative leakage area around the perimeter of small, medium, and large masks with the tuck-in ratio of *α*_*T*_ = 0.7, 0.5, 0.3. The bars are for the CDC recommended mask (medium size) while the blue and red dots represent large and small masks, respectively. Each category of faces are plotted with a different color and the tuck-in ratios are shown with bars with solid (*α*_*T*_ = 0.7), dashed (*α*_*T*_ = 0.5) and dotted (*α*_*T*_ = 0.3) borders. The error-bar for each data shows the standard deviation of the computed parameters for the 150 random face realizations. It is found that the smaller mask size, relative to the CDC recommended size, has minimal effect on the total leakage area for the base cases, especially for higher tuck-in ratios. However, there are substantial changes in the total leakage area for thinner, younger and more feminine faces with a decrease in mask size regardless of the tuck-in ratio. In general, the trend seems to indicate that smaller masks will reduce the area across the spectrum of faces. Similarly, the total leakage area continuously reduces with more tuck-in on sides for all but the heavy face category. The side-edge tuck-in has a more pronounced effect on the large mask size. Later we show how the large mask is oversized for some of the cases, and this oversized mask hangs off the chin instead of fitting snug against the face. Tucking in (pleating) the sides of the mask shifts the lower edge of the mask closer to chin and therefore significantly reducing the gap area.

The leakage around the edges of the mask is also dependent on the hydraulic perimeter of the opening (both area and shape of opening) [36, 37]. To explore this effect, in Fig 5b we show the maximum opening (maximum distance between the mask edge and face). Surprisingly, the reduction of the mask size does not have a monotonic effect on the maximum gap opening (Fig 5b). That is, while the majority of face categories show some reduction in the maximum opening, the base, heavy, and masculine faces can have a larger maximum opening with smaller masks. Only the thin, feminine, and short faces show a significant improvement in maximum opening with a reduction in mask size from the CDC recommended size. The maximum opening also reveals that the tuck-in ratio does not have a universal effect. As an example, smaller tuck-in ratios result in larger openings in older faces, albeit a small increase. The main observation is that proper mask sizing is the most effective way to reduce the maximum opening and a smaller tuck-in ratio could only be beneficial in certain categories of faces.

**Fig 6:**
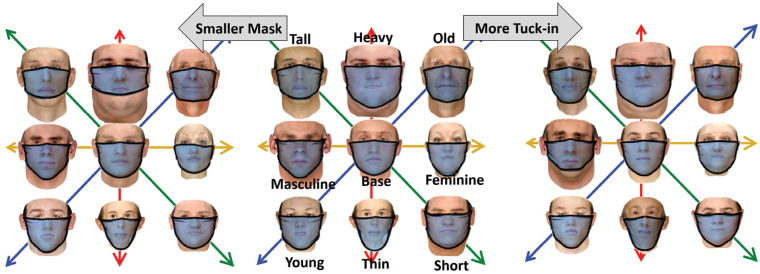
The middle plot are the median cases for the medium mask (CDC recommended size) and tuck-in ratio of 0.5. Right and left plots show the modification for the small mask and tuck-in ratio of 0.3, respectively.

The comparisons between mask sizes and tuck-in ratios for the median cases in each category are shown in Fig 6. It can be seen that the placement of the mask on the face is greatly modified when the mask becomes smaller than a threshold. In particular, the lower edge of the mask shifts from below the chin to the top of the chin for heavy and tall faces with a small mask. Consequently, the bottom support of the mask can slip easily in tangential directions and could easily lead to changes in the mask placement during routine daily activities such as talking and breathing. Any case that exhibits mask slippage, whether the bottom edge slipping past the chin support point or the top edge slipping below the nose tip point, are considered failures and were not used in evaluating any of the metrics in this study. We note that the small mask on heavy and tall faces failed for the majority of cases. The results presented are for the cases where the mask did not slip, however, due to the majority of cases with small masks in these particular cases (heavy and tall) failing, we disregard them in the rest of our discussion. The thin and feminine cases exhibit a rapid increase in the opening gap, primarily in the chin area, with an increase in the mask size. The results suggest that the addition of a tuck-in mechanism to the lower edge of the medium size mask is a simple modification that would make them more effective for feminine and thin faces.

**Fig 7:**
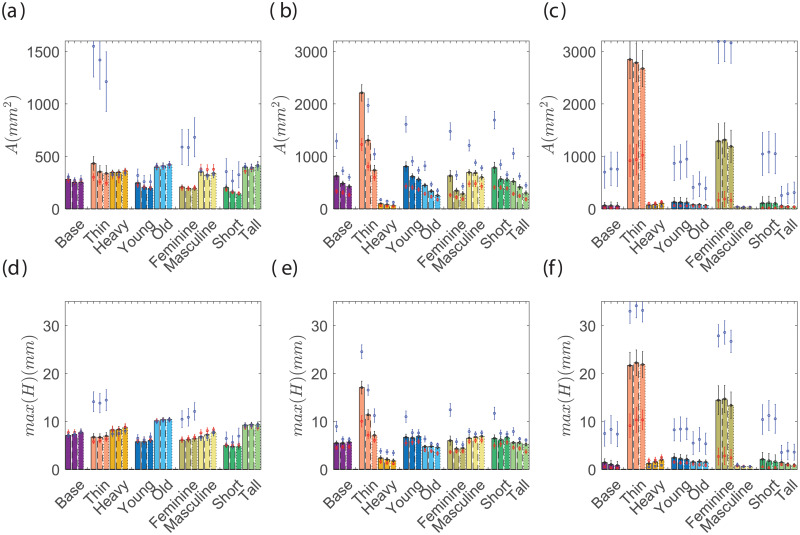
Average perimeter opening area (a-c), and maximum opening gap (d-f) in three edges of the mask. The description of the data is similar to Fig 5.

The leakage around the mask can be divided into three distinct segments: the top edge near the nose, the side edges near the cheeks, and lower edge near the chin. The maximum gap opening and the contribution of each segment of the mask to the total leakage area are compared in Fig 7. It is observed that the leakage from the nose area is independent of the tuck-in ratio and mask size, except for thin, feminine, and short faces (i.e. smaller faces). When the mask is medium or small, the tuck-in ratio can be used to further reduce the opening near the nose (side 1) in thin, young, and short faces without increasing the maximum gap distance. For the other cases, more tuck-in is accompanied by an increase in the maximum gap opening on top of the mask. The increase is primarily due to changes in the placement of the mask on the nose. The insensitivity to mask size and tuck-in ratios from the majority of the face groups suggests that a clip or other mechanical effect is necessary to reduce the top edge opening, something that is standard in some higher quality masks currently in the market.

A different trend is observed for the cheek edges (side 2) where there is a substantial reduction of opening with a decrease in mask size and tuck-in ratio. The maximum opening is significantly reduced by increasing the tucking from *α* = 0.7 to *α* = 0.5 in large masks, further increase in tuck-in (lower *α*) does not change to the maximum opening. The leakage area from this side shows the greatest reduction with more tuck-in relative to the other two regions of the mask. The reduced area and unchanged maximum opening indicate that with smaller tuck-in ratios, the side opening gap opening becomes more concentrated.

The maximum opening and the leakage area near the chin (side 3) are major components of the total leakage for thin and feminine faces. The tuck-in ratio has an insignificant role in this part of the mask, while the mask size is the primary driving factor. Thin and feminine faces show a more than 50% reduction in leakage area with the smaller than the CDC recommended mask size. An exception is the heavy faces, where a small mask induces larger maximum openings in the lower edge. This is due to the face-mask slip on the face and placement of the lower edge on top of the chin area.

**Fig 8:**
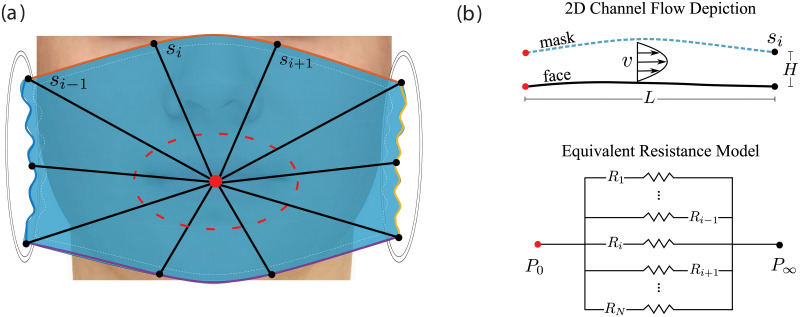
a) Two dimensional channels depicted on the mask deployed on a face by solid black lines emanating from the high pressure region at the mouth and terminating at the edge of the mask. b) Schematic of pressure driven channel flow corresponding to the points along the perimeter of the mask (*s*_*i*_) and the relevant circuit.

It is apparent that the leakage area (*A*) or maximum gap (*max*(*H*)) alone are not perfect indicators of the mask’s effectiveness. Instead, we propose looking at the mask as a set of *N* channels with one end at the mouth/nose (i.e., the region at which a high pressure is generated that will drive the air towards the perimeter of the mask), and the other end at the outer edge of the mask. This can be visualized as rays emanating from the mouth to points along the mask edge (*s*_*i*_), as shown in Fig 8a. These rays can be thought of as two-dimensional channels of length *L* and height *H*, equal to that of the mask opening at each point along the perimeter of the mask. Fig 8b shows such a channel, note that the length of each channel is given by the distance from the mouth to the corresponding point on the mask perimeter (*s*_*i*_). Considering the system in this way, we can derive a hydraulic resistance corresponding to each point along the perimeter of the mask(*R*_*i*_), which would describe the relative amount of the airflow that leaks out at this point compared to the amount filtered out through the mask cloth. The velocity profile for permeable channel flow can be defined as,

$$\begin{array}{c} v\left(x,y\right)=\frac{H^{2}}{2\mu }\frac{\partial p}{\partial x}\left(\frac{y}{H}-\frac{y^{2}}{H^{2}}\right). \end{array}$$ (11)

Note that although one side of the channel is porous, i.e. the mask, it has been shown that the velocity profile does not change significantly and therefore, the Hagen-Poiseuille flow profile is still valid for our analysis [38]. Upon integrating *v* across the height of the channel, the mass flow rate can be obtained as,

$$\begin{array}{c} \dot{m}=\frac{H^{3}}{12\mu }\frac{\Delta p}{L}, \end{array}$$ (12)

where Δ*p* = *P*_0_ − *P*_∞_, the difference between the high pressure near the mouth and ambient pressure just outside the mask. From this, an equivalent resistance model as seen in Fig 8b can be used to derived a hydraulic resistance for each point along the mask perimeter as,

$$\begin{array}{c} R_{i}=\frac{\Delta p}{{\dot{m}}_{i}}=12\mu \frac{L_{i}}{H_{i}^{3}}. \end{array}$$ (13)

Only *L* and *H* are geometrically varying parameters in this expression; therefore, we introduce a new parameter ${\bar{R}}_{i}=\frac{L_{i}}{H_{i}^{3}}$ to characterize the relative changes in the hydraulic resistance. Since $\bar{R}$ is inversely proportional to the leakage mass flow rate, a larger $\bar{R}$ is viewed as more effective.

**Fig 9:**
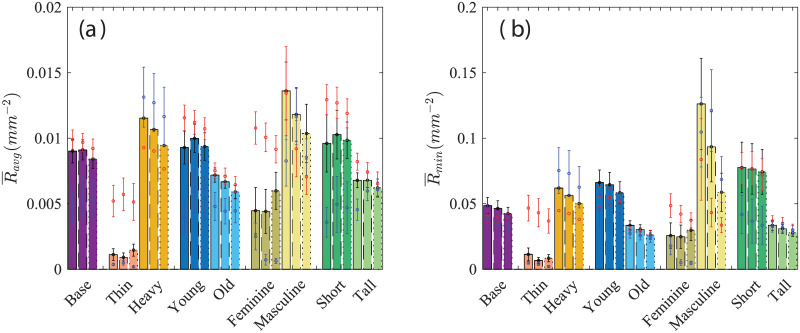
(a) Average hydraulic resistance *R*_*avg*_ and (b) the minimum hydraulic resistance *R*_*min*_ for different mask sizes and tuck-in ratios. The description of the data is similar to Fig 5.

The average and minimum of $\bar{R}$, for each face category and mask design, are shown in Fig 9. The average $\bar{R}$ is taken as the average over all cases for each face category and mask, of the total resistance of each case. As shown in Fig 8b, all channels, and therefore also the resistances, are in parallel such that,

$$\begin{array}{c} {\bar{R}}_{avg}=mean({\left(\sum_{i}\frac{1}{{\bar{R}}_{i}}\right)}^{-1}). \end{array}$$ (14)

Contrary to the previous metrics *A* and *max*(*H*), the mean hydraulic resistance *R*_*avg*_ does not show that smaller masks are generally a better choice. Instead, there is a more clear distinction between the most effective mask design for each face category. The largest mask provides the highest *R*_*avg*_ for heavy and tall faces. Base, young, old, and masculine faces benefit the most from the medium mask, while the rest of the faces attain the best protection in terms of *R*_*avg*_ with the small mask. As noted previously with *A* and *max*(*H*) any mask larger than the small mask on thin faces is mostly ineffective due to the large gaps especially in the overhanging lower edge. The hydraulic resistance accounts for the proximity of the mask edge to the mouth/nose such that if a smaller mask cause slippage that leads to a reduction in the distance between the mask edge and mouth/nose, the hydraulic resistance will decrease. Interestingly, the non-monotonic effect of the tuck-in ratio is more apparent in *R*_*avg*_. Tuck-in seems to provide the necessary adjustments to provide the most effective mask for several cases. Young faces show that smaller masks are more effective for their face, however it seems that a medium mask with tuck-in can be the better choice. Masculine and short faces, show similar behavior. In the base case, for example, increasing the tuck-in ratio is detrimental to the overall effectiveness of the small mask. As the mask size is increased, the larger tuck-in ratios perform better. This trend is clearly not monotonic, instead *R*_*avg*_ initially increases with *α* but increasing beyond *α* = 0.5 results in a decrease in *R*_*avg*_. To understand this we take a closer look at the masculine faces with the large mask where the effect is most noticeable. It is found that increasing the tuck-in ratio from *α* = 0.3 to *α* = 0.5 results in a decrease in gap area and maximum gap opening for all sections of the mask resulting in higher hydraulic resistance. Increasing to *α* = 0.7 results in an increase in the maximum gap opening. This translates to a change in the shape of the openings from wide and shallow to more localized larger gaps accounting for the decrease in hydraulic resistance in large masks and large tuck-in. The effectiveness of the mask could also be defined by the most likely point of leakage, here defined by the point with the lowest hydraulic resistance (*R*_*min*_). While there are minor differences in the trends, they are mostly insignificant. The most notable difference is that *R*_*min*_ is in the short faces where the medium mask seems to be more effective than the small mask, contrary to the observations made in *R*_*avg*_.

**Fig 10:**
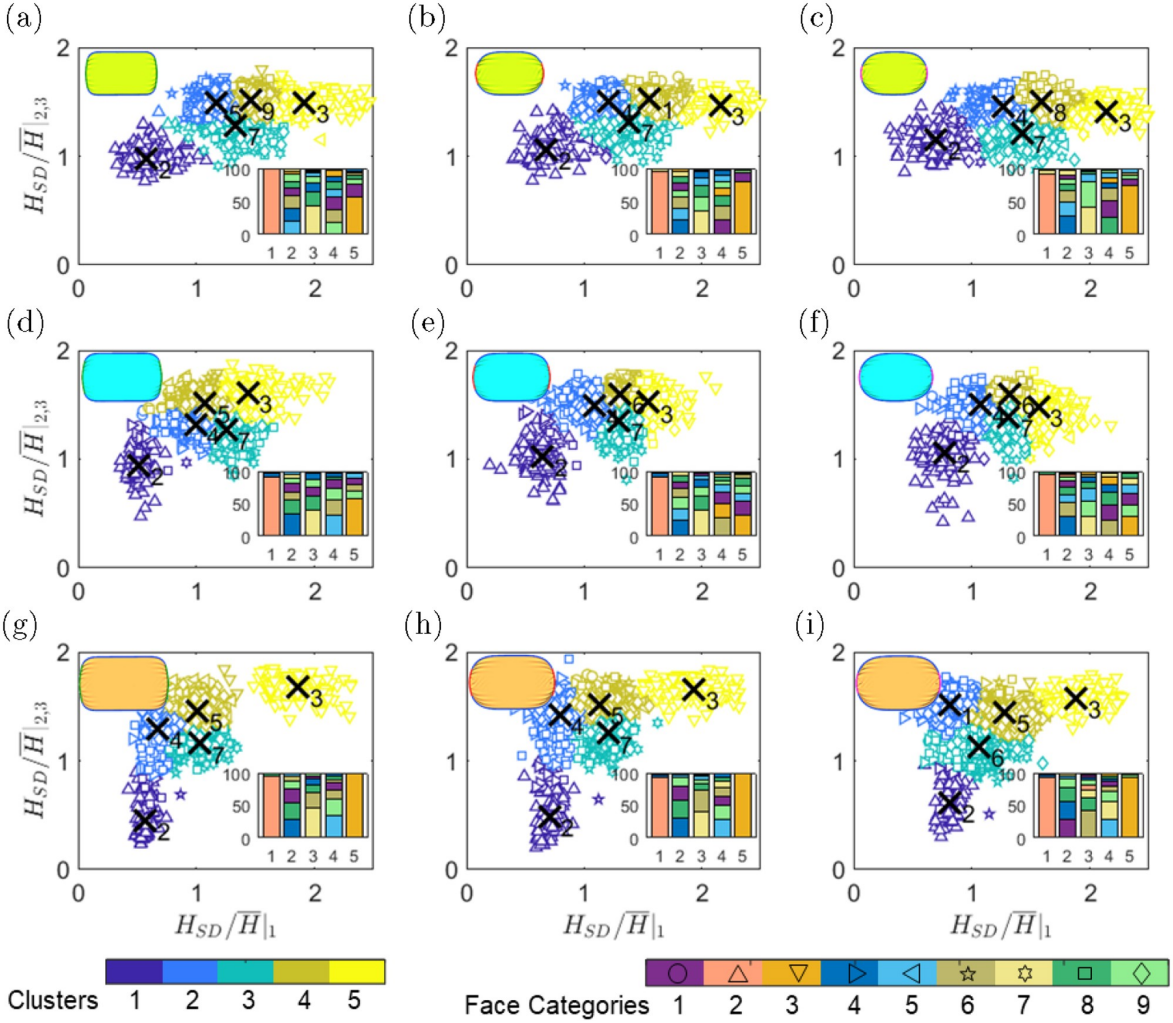
Classification of the leak for different mask size (rows) and tuck-in ratio (columns) based on the shape parameter of $H_{SD}/\bar{H}$. Clusters are displayed with different colors and different face categories are marked with symbols given in the legend. The inset figures represent the percentage of constituent faces in each category.

The previous results show that both leakage area and maximum gap opening of the edges should be considered to reach a discriminatory factor that can identify different modes of leakage around the mask. Toward this, we found that $H_{SD}/\bar{H}$ can serve as the parameter for unsupervised classification of the results, where *H*_*SD*_ is the standard deviation of the opening gap and $\bar{H}$ is the average opening distance along the edges. Fig 10 shows the scatter plot for mask size and tuck-in ratio effects. It is found that the data can be grouped into 5 clusters to best separate the effect of facial features. The number of clusters (*K*) is chosen such that with a further increase of *K*, the reconstruction error is not significantly reduced. The reconstruction error is defined as $E\left(D,K\right)={\left|D\right|}^{-1}\sum_{i\in D}{∥x_{i}-\mu_{z_{i}}∥}^{2}$, where D is the data set and **μ** is the center of the cluster $z_{i}\;=\;{\text{argmin}}_{k}{∥x_{i}-\mu_{k}∥}_{2}^{2}$. These clusters are marked with different colors in each sub-figures, while different symbols are used to distinguish between different feature categories. Besides, the inset figures present the percentage of face categories in each cluster.

The thin face category (number 2 in the legend) is consistently the primary contributor of cluster 1 (dark blue cluster). Similar observations can be made for heavier faces and cluster 5 (yellow cluster). The center of cluster 1 shifts to higher $H_{SD}/\bar{H}{|}_{1}$ and $H_{SD}/\bar{H}{|}_{2,3}$ with decreasing tuck-in ratio, consistent with previous observations that a smaller tuck-in ratio leads to more non-uniform gap distributions. An increase in mask size shifts the cluster centroid to lower $H_{SD}/\bar{H}{|}_{2,3}$, indicating that the mask size will primarily affect the gap opening on lower and side edges. Cluster 5 only shows variations along the $H_{SD}/\bar{H}{|}_{1}$ axes. Also, we see that the masculine face category is the most prevalent member of cluster 3, with a minor centroid shift among the cases. The other two clusters, clusters 2 and 4, are mixed sets of faces suggesting that other facial features are needed to classify this region of the features sub-space. It is found that the tuck-in ratio can only induce a minor shift to these clusters, but the mask size can substantially modify the mode of opening along the edges.

**Fig 11:**
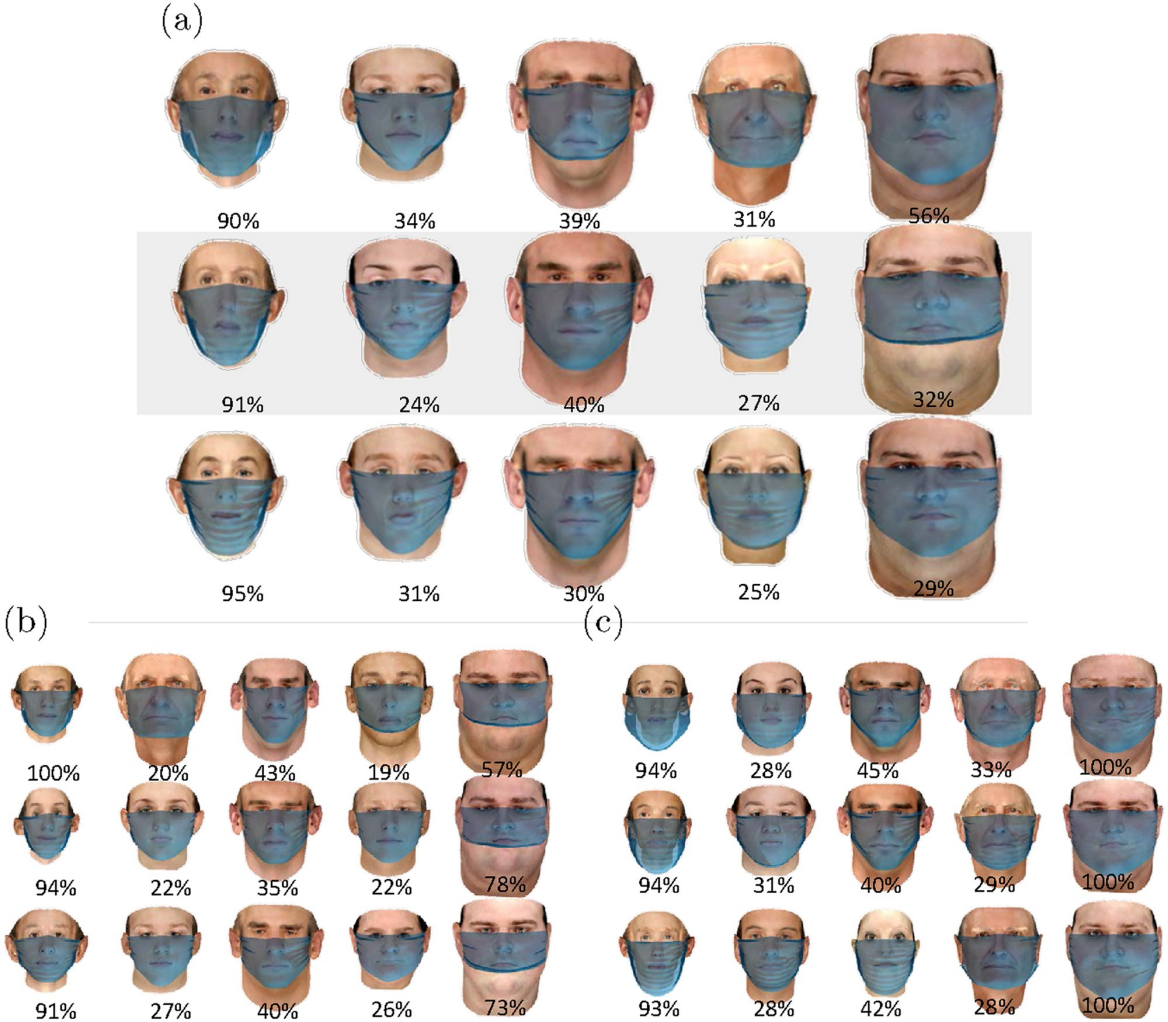
The nearest cases to the center of the clusters and the percentage of them in each cluster for (a) medium(CDC recommended) mask, (b) small mask, and (c) large mask for three tuck-in ratios of 0.7, 0.5 and 0.3 from the top row to bottom.

In Fig 11, we plot the cases of the dominant feature category nearest the cluster centroids of Fig 10 for medium (a), small (b), and large (c) mask sizes. Each figure also include the results for the tuck-in ratios of 0.7 (top row), 0.5 (middle row) and 0.3 (bottom row). As mentioned previously, clusters 1, 3, and 5 are predominantly comprised of thinner, more masculine, and heavier faces, respectively. The young and short faces are the most representative feature categories of cluster 2, while the old and feminine faces form the majority of cluster 4 for medium and large masks. Nonetheless, it is found that none of the feature categories is the dominant constituent of clusters 2 and 4 with more than 1/3 of the members.

The results from unsupervised clustering based on the face-associated variabilities suggest that for certain groups such as heavy and thin faces, it is possible to find the most effective face covering with minimal gap opening, especially with the use of proper mask size. However, for the other cases, one needs to consider other factors such as shape and geometrical parameters of the face to better identify the most optimal cloth mask covering size and tuck-in ratio.

### 3.2 Role of facial features

**Fig 12:**
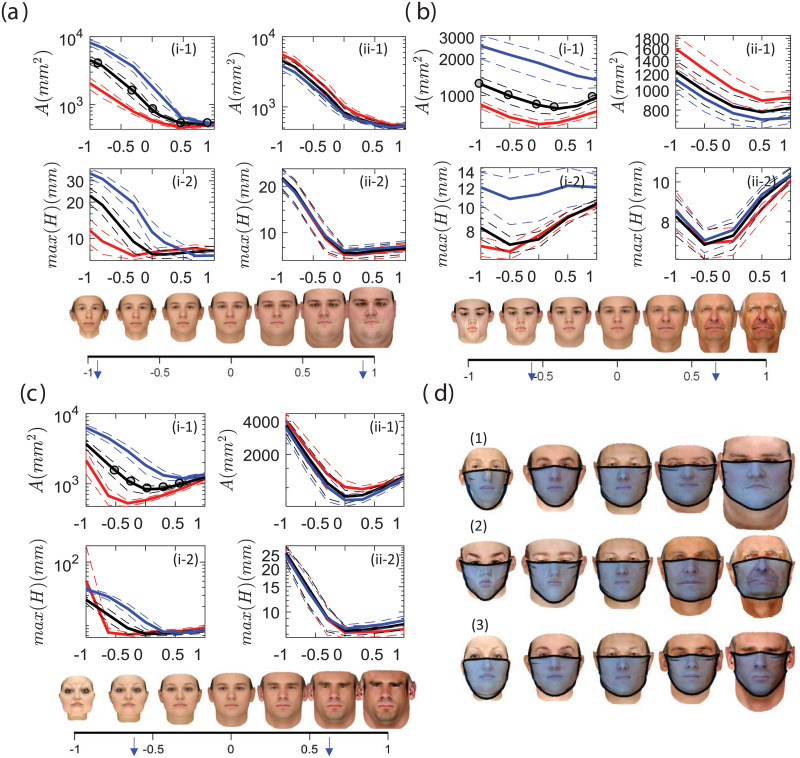
The changes in the leakage area (part 1), maximum gap opening (part 2) for different (a) weight, (b) age and (c) gender indices. Panel (i) is for 3 mask sizes (red: small, black:medium, and blue: large) with tuck-in ratio of 0.5, and panel (ii) is for 3 tuck-in ratio (red:0.7, black:0.5 and blue:0.3) and the medium mask size. The x-axis is the normalized feature mode shown for the average face in the subset figures. The median cases for the medium mask size and tuck-in ratio are 0.5 marked in the subfigures i-1.

This section explores how the changes in the categorical facial features (weight, age, and gender) affect the findings presented. The leakage area and maximum gap distance are shown in Fig 12. The horizontal axis is hereafter named as weight/age/gender index, with a zero value corresponding to the base case. The associated facial feature for each value is depicted in the legends, and the cases used in the previous sections are marked with. The left column is for different mask sizes and the tuck-in ratio of 0.5 and the right column is for the medium (CDC recommended) mask size with tuck-in ratios of 0.3, 0.5, and 0.7. The dash lines are showing the standard deviation of data. Finally, in Fig 12d we plot the median cases for the marked dots in the sub-figures (i-1) for the medium mask and the tuck-in ratio of 0.5.

The increase in the weight feature results in decaying leakage area until a threshold at which the leakage area reaches an asymptotic value. This threshold is very similar between different mask sizes and happens at a weight index of 0.5 (Fig 12a-i). The weight index with the minimum gap is highly dependent on the mask size, observed at -0.3, 0, and 0.6 for the small, medium, and large masks respectively. On the other hand, the higher tuck-in ratio marginally reduces the opening area for all weight indices and has almost no effect on the minimum opening gap (Fig 12a-ii panel). As shown in Fig 12d-1, the gap opening along the bottom edge of the mask changes most significantly and its tightness on the chin is correlated with the transition point observed above.

The change in the age feature of the face has a different outcome on the leakage area and maximum gap opening. Both the tuck-in ratio and mask size almost equally modify the leakage area and maximum opening with the age index (Fig 11b). The minimum leakage area with respect to the age index shifts to older faces with increasing mask size but is not affected by the tuck-in ratio. The large mask shows almost similar maximum opening across all ages, while the other sizes show initial decay and subsequent rise with age index. The smallest maximum opening occurs at a lower age index than does the smallest leakage area. The tuck-in ratio further changes the maximum opening, especially at the low and high extremes of the age index. The median realizations for highlighted cases in panel (i-1) in Fig 11d-2 indicate that there is a shift in the location of the upper edge of the mask on the nose with the age index. A simple mask design is found to be incapable of reducing the top edge opening near the nose as the geometric dissimilarities between the mask and the face always result in a non-zero gap at the top edge.

The gender index, while showing a similar trend in total leakage area to the age index, has a distinct maximum opening profile, which is more similar to that of the weight index (Fig 11c). The minimum leakage area is shifted to more feminine faces with smaller mask sizes, but the maximum opening becomes larger with the increase of the feminine gender index. In fact, the smallest mask exhibits the largest gap opening than any other case when the gender index is -1 (most feminine). For more masculine faces, the response of all mask sizes is similar. Finally, the tuck-in ratio has negligible effects across the gender index. From the tested cases, it is found that all gender faces have similar gap distributions in cheek and chin areas, but they are different in the opening at the nose area compared to other indices like weight.

## 4 Discussion

### 4.1 Summary

The findings from the previous sections illustrate that it is important to account for a wide representative population of faces whenever a mask fit/design study is performed. It is shown that cases with smaller facial dimensions such as thinner, younger, and more feminine faces, tend to suffer from more leakage due to improper fit of homemade cloth masks. There is, in fact, a threshold in mask size at which these faces show a significant increase in leakage area, especially from the bottom edge of the mask. Based on the depiction of the median cases (Fig 12d), it is observed that this increase is due to an oversized mask hanging off the face near the chin. Of the three mask sizes studied, all but heavy and masculine faces showed the reduced leakage area with the smaller masks. In some cases reducing the leakage area by over 50%, compared to the CDC recommended size. Although the total leakage area was reduced with smaller masks for most faces, the smaller masks do not extend below the chin for all face types (heavier and more masculine). This, of course, can increase the risk of new perimeter leaks during routine daily activities like breathing and talking, and especially during high transmission actions such as sneezing and coughing. The other simple modification to masks, besides the size, is the tuck-in of the side edges of the mask. In general, larger tuck-in (smaller tuck-in ratio) leads to reduced leakage areas. However, we know at least intuitively, that small masks are not the most effective for all faces.

The effective hydraulic resistance is proposed as a more discriminatory metric that considers the gap along the perimeter of the mask and the distance from the mouth (source of aerosols). The hydraulic resistance shows a clearer distinction between the most effective mask between face categories. Smaller does not seem to be better as indicated by *R*_*avg*_. Thin, female, and short faces showed the smallest mask to be the most effective. Base, young, old, and male faces had the highest hydraulic resistance with the medium mask, while heavy and tall faces did best with the largest mask. Although not explicitly clear, the hydraulic diameter accounts for the shift of the mask lower on the nose for smaller masks. The shift of the mask on the nose reduces the distance from the mouth to the outer edge reflected in the hydraulic diameter. It is clear from Fig 9a that even simple mask design elements, such as mask size and tuck-in ratio, have significantly different effects on each face type, and further, that the combined effects of these design elements are not easily predicted.

As mentioned in section 3.1, the shape of the opening is also crucial in determining the effectiveness of a mask, especially for outward protection. We describe this with the maximum gap opening *max*(*H*). Given the same leakage area, the mask that produces larger *max*(*H*) has more localized openings as opposed to the mask with smaller *max*(*H*) would have a more uniform slit-like opening through the length of the mask edge. During a respiratory event, the more localized openings would create higher exit velocity jets, which would further spread the aerosols. From this, we cannot definitively conclude that masks that reduce leakage areas are best. Instead, we must also ensure that the reduction in leakage area is not accompanied by an increase in *max*(*H*). This effect is also present indirectly in the hydraulic resistance, and hence is the reason why we see the difference in optimal mask design for each face type.

A deeper study of key features, such as weight, age, and gender further highlighted the non-monotonic effects of the explored design elements. The direct effect of the weight-dependent facial features shows that after a weight-index of 0.5, mask size and tuck-in have no effect on the leakage area. Heavier faces were observed to have more uniform openings (smaller maximum gap) with the larger masks but this was much dependent on the weight index. The same analysis was carried out on the age and gender feature categories. Both of these saw, by and large, a decrease in leakage with smaller masks across the feature’s index. Masculine faces, similar to heavier faces, show negligible effects of both mask size and tuck-in ratio. The feminine faces did show some reduced leakage area when the mask is small but interestingly, the small mask produced the largest maximum gap opening in the most feminine faces (index of -1). There are optimal values for both the age and gender indices where the leakage area and maximum opening attain their minimum values. This would lead one to believe that there are parameters other than the feature categories explored here, on which the mask fit depends.

### 4.2 Strengths and limitations

Several limitations are present in this study. The design elements in masks are numerous and only two simple design elements were discussed. This, of course, was a necessary step to reduce the size of this study. However, the framework proposed can be easily extended to account for any other mask designs, including varying geometries, stitch patterns, and mechanical devices. Another limitation is related to the static nature of the model. It is known that during violent respiratory events such as coughing, the mask can deform due to the pressure build-up inside the mask and therefore affect the efficacy. The flow speeds during inspiration and expiration phases also differ. It is anticipated that lower pressure in the region interior to the face-mask during inhalation could induce inward deformation to the mask and reduce the perimeter leaks. On the other hand, the higher pressure during the expiration process might create larger leakage openings and depend on the instantaneous shape of the mask, induce stronger or weaker leakage jets on sides. Activities such as speaking can also cause the mask to shift and deform, affecting the efficacy of the mask.

The 3D morphable face model accounts for large sample sizes of subjects with many different facial features at scales not achievable by experimental methods. It also serves to systematically study independent characteristics such as the shape and size of the nose and jaw, or macro features such as was done here in 3.2. The entire framework is flexible enough to allow fast exploration of many mask designs, providing us with a powerful tool in developing more effective and comfortable masks.

## 5 Conclusion

The effect of mask fit for a large cohort of individuals with varying facial features was studied using three-dimensional, morphable, headform models onto which a cloth mask was deployed via a quasi-static simulation. The categorical study of facial features (weight, age, gender, height) prove that the CDC recommended mask size is perhaps not the most effective mask size for the entire population. Thin, young, feminine, and short faces were observed to benefit from a smaller mask size. Heavier and taller faces, on the other hand, would benefit from a larger mask. For the base, masculine, and older faces, the best performance is achieved with a medium mask. More importantly, although tuck-in of the side-edges can reduce the leakage area, it can, in turn, cause larger gaps. The effect of the tuck-in ratio was observed to be more effective on larger masks. However, the tuck-in ratio has a non-monotonic behavior that changes for each subject and the mask size. It is apparent that it would be nearly impossible to have one universal recommendation for all subjects based solely on the feature vectors in this study, alluding to important topological features that can significantly impact the mask fit. It furthers highlights the necessity of approaching the task of mask design in a statistically inclusive way that accounts for the large variation in the population of faces. It is also found that the effects of the mask design elements are not easily predictable and need to be characterized on a more individual basis.

The total leakage area does not reveal the complete picture of mask effectiveness. The examination of leakage area by section shows the principle sections of worry for each category of faces. The total leakage area in thinner and feminine faces predominantly comprises the opening near the chin due to oversized mask sagging below the chin. A lower edge tuck-in modification could reduce the leakage near the chin. For the remainder of faces, except the heavier and more masculine faces, the cheek opening is the major component of the total leakage area and can be reduced with tuck-in. Finally, the top edge opening in all subjects is mostly unaffected by the mask size and tuck-in, suggesting that other mechanical means are necessary to reduce the leakage in this section. For a more discriminatory metric to determine the best mask for each face, hydraulic resistance was introduced. Analysis about the flow of respiratory events needs to be carried out to arrive at a definitive conclusion on the most appropriate metric to quantify efficacy.

As a future direction, more facial features and race should be included in the population-based study as well as the comfort factor. More design elements should also be explored including mechanical nose clips, and different mask geometries.

## Supporting information

S1 Dataset Mask fit metrics. Data files corresponding to the figures in this paper are provided as a Zip file. The data is archived as a Matlab.mat file and the description of the data is included in the archive file.
(ZIP)
